# Persistent endothelial dysfunction in post-COVID-19 syndrome and its associations with symptom severity and chronic inflammation

**DOI:** 10.1007/s10456-023-09885-6

**Published:** 2023-07-28

**Authors:** Timon Kuchler, Roman Günthner, Andrea Ribeiro, Renate Hausinger, Lukas Streese, Anna Wöhnl, Veronika Kesseler, Johanna Negele, Tarek Assali, Javier Carbajo-Lozoya, Maciej Lech, Heike Schneider, Kristina Adorjan, Hans Christian Stubbe, Henner Hanssen, Konstantin Kotliar, Bernhard Haller, Uwe Heemann, Christoph Schmaderer

**Affiliations:** 1 School of Medicine, Klinikum Rechts Der Isar, Department of Nephrology, Technical University of Munich, Ismaninger Str. 22, 81675 Munich, Germany; 2 Faculty of Health Care, Niederrhein University of Applied Sciences, Krefeld, Germany; 3 Medizinische Klinik Und Poliklinik IV, LMU University Hospital Munich, Ziemssenstraße 5, 80336 Munich, Germany; 4 School of Medicine, Klinikum Rechts Der Isar, Department of Clinical Chemistry and Pathobiochemistry, Technical University of Munich, Ismaninger Str. 22, 81675 Munich, Germany; 5 Department of Psychiatry and Psychotherapy, LMU University Hospital Munich, Nußbaumstraße 7, 80336 Munich, Germany; 6 Medizinische Klinik Und Poliklinik II, LMU University Hospital Munich, Marchioninistraße 15, 81377 Munich, Germany; 7 Department of Sport, Exercise and Health, Preventive Sports Medicine and Systems Physiology, University of Basel, Basel, Switzerland; 8 Aachen University of Applied Sciences, Heinrich-Mussmann-Str. 1, 52428 Jülich, Germany; 9 School of Medicine, Institute for AI and Informatics in Medicine, Technical University of Munich, Klinikum Rechts Der Isar, Ismaninger Str. 22, 81675 Munich, Germany; 10 German Centre for Infection Research (DZIF), Partner Site Munich, Munich, Germany

**Keywords:** Endothelial dysfunction, Long COVID, Post-COVID-19 syndrome, Retinal microvasculature

## Abstract

**Background:**

Post-COVID-19 syndrome (PCS) is a lingering disease with ongoing symptoms such as fatigue and cognitive impairment resulting in a high impact on the daily life of patients. Understanding the pathophysiology of PCS is a public health priority, as it still poses a diagnostic and treatment challenge for physicians.

**Methods:**

In this prospective observational cohort study, we analyzed the retinal microcirculation using Retinal Vessel Analysis (RVA) in a cohort of patients with PCS and compared it to an age- and gender-matched healthy cohort (*n* = 41, matched out of *n* = 204).

**Measurements and main results:**

PCS patients exhibit persistent endothelial dysfunction (ED), as indicated by significantly lower venular flicker-induced dilation (vFID; 3.42% ± 1.77% vs. 4.64% ± 2.59%; *p* = 0.02), narrower central retinal artery equivalent (CRAE; 178.1 [167.5–190.2] vs. 189.1 [179.4–197.2], *p* = 0.01) and lower arteriolar-venular ratio (AVR; (0.84 [0.8–0.9] vs. 0.88 [0.8–0.9], *p* = 0.007). When combining AVR and vFID, predicted scores reached good ability to discriminate groups (area under the curve: 0.75). Higher PCS severity scores correlated with lower AVR (*R* = − 0.37 *p* = 0.017). The association of microvascular changes with PCS severity were amplified in PCS patients exhibiting higher levels of inflammatory parameters.

**Conclusion:**

Our results demonstrate that prolonged endothelial dysfunction is a hallmark of PCS, and impairments of the microcirculation seem to explain ongoing symptoms in patients. As potential therapies for PCS emerge, RVA parameters may become relevant as clinical biomarkers for diagnosis and therapy management.

**Trial registration:**

This study was previously registered at ClinicalTrials (“All Eyes on PCS—Analysis of the Retinal Microvasculature in Patients with Post-COVID-19 Syndrome”. NCT05635552. https://clinicaltrials.gov/ct2/show/NCT05635552).

**Graphical abstract:**

Persistent endothelial dysfunction in post-COVID-19 syndrome. Acute SARS-CoV-2 infection indirectly or directly causes endotheliitis in patients. *N* = 41 PCS patients were recruited and retinal vessel analysis was performed to assess microvascular endothelial function. Images of SVA and DVA are illustrative for RVA data analysis. For each PCS patient and healthy cohort, venular vessel diameter of the three measurement cycles was calculated and plotted on a diameter-time curve. Patients exhibited reduced flicker-induced dilation in veins (vFID) measured by dynamic vessel analysis (DVA) and lower central retinal arteriolar equivalent (CRAE) and arteriolar-venular ratio (AVR) and a tendency towards higher central retinal venular equivalent (CRVE) when compared to SARS-CoV-2 infection naïve participants. Created with BioRender.com

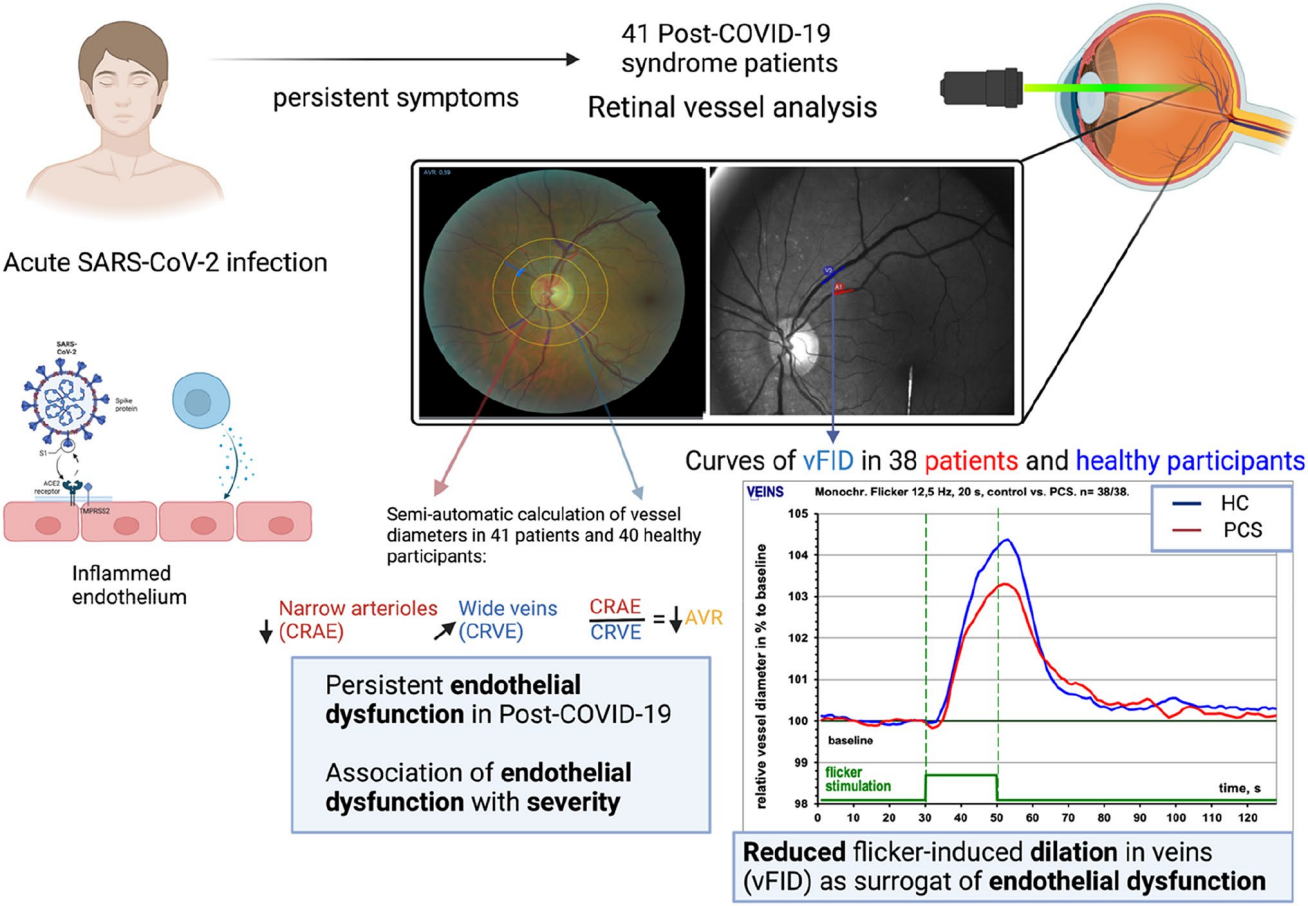

**Supplementary Information:**

The online version contains supplementary material available at 10.1007/s10456-023-09885-6.

## Introduction

Between 10 to 35% [1–3] SARS-CoV-2 infected patients have ongoing symptoms such as shortness of breath, chest pain, cognitive dysfunction, fatigue and palpitations. This constellation of symptoms is commonly referred to as post-COVID-19 syndrome (PCS, also known as Long COVID) if one or more of these symptoms persist for more than two months and cannot be explained by any other diagnosis [4–6]. PCS can lead to a health-related decline in quality of life and interferes with daily life and activity [7, 8]. Patients may develop comorbidities such as anxiety disorders, depression and cognitive symptoms, reducing the likelihood of employment and working full time [9, 10], highlighting the socioeconomic impact of PCS.

Understanding the pathophysiology behind PCS better and developing tools to measure disease severity objectively are critical objectives of ongoing research. Several hypotheses have been developed regarding the pathophysiology of PCS, with microthrombus formation, autoimmunity, and viral persistence among the most discussed [11].

Acute SARS-CoV-2 infection leads directly and indirectly to endothelial dysfunction (ED) [12]. Subsequently, markers of inflammation and ED are elevated during acute infection. This can lead to unresolved chronic inflammation in PCS patients, with elevated levels of C-X-C motif chemokine ligand 10 (CXCL10), interleukin-6 (IL-6), and D-dimer even months after the acute phase of infection [13–16]. Vascular abnormalities in PCS patients have been observed, characterized by a persistent rarefaction and limited responsiveness to local variations in metabolic demand [17]. Low-grade inflammation may explain persistent ED in PCS patients and even though the exact mechanism of direct SARS-CoV-2 infection of the endothelium remains unclear, studies using optical coherence tomography (OCT) and flow-mediated dilation (FMD), as well as indirect measurements of serum biomarkers of ED, suggest persistent endotheliopathy following acute infection [16, 18–20]. The few studies that have evaluated persistent ED in PCS and its association with symptoms were mostly limited to medium to large-sized arteries using peripheral arterial tone (PAT) or flow-mediated dilation (FMD) technology or indirectly measuring biomarkers of endothelial damage [20–22]. Although evidence grows that persistent ED in large-sized peripheral arteries may contribute to ongoing symptoms in PCS patients, little is known about ED in the microvasculature of PCS patients. An estimated 90% of endothelial cells are found in the microcirculation, which makes it the ideal vascular bed for quantifying potential endotheliopathy [23].

Dynamic vessel (DVA) and static retinal vessel analysis (SVA) are two well-established diagnostic tools to analyze retinal microcirculation as a surrogate of pan-endothelial health [23, 24]. Assessing retinal endothelial function has been proposed as “of high potential” to quantify ED non-invasively by the European Society of Cardiology (ESC) [25]. DVA measures retinal vessel responses to flickering light over time, which is mediated by neuro-vascular coupling and subsequently flow-induced nitric oxide (NO) release from the endothelium [23]. SVA offers an accurate, quick, and reproducible method to assess impairment of microvascular integrity and vessel morphology by measuring vessel diameters. SVA and DVA have both been used and proven as valuable diagnostic tools to determine endothelial health in large cohorts with chronic cardiovascular (CV) diseases [26–29]. The Atherosclerosis Risk In Communities (ARIC) study showed that narrower central retinal arteriolar equivalent (CRAE) and wider central retinal venular equivalent (CRVE) are independent predictors for long-term CV events in a large cohort of healthy participants [26, 28]. We previously could show in a cohort of dialysis patients that impaired retinal venular dilation (vFID) is an independent predictor for all-cause mortality [30].

So far, data on DVA and SVA are missing in PCS patients. As patients experience ongoing cardiovascular and neurovascular symptoms, we hypothesize, that endothelial function quantified by RVA might be key for better understanding prolonged symptoms in PCS.

## Materials and methods

### Study design and cohort

The present study is part of the "All Eyes on PCS" study, which is a prospective, observational single-center study examining the retinal microvasculature of PCS patients and providing an in-depth clinical characterization of the patients. The study protocol was approved by the local ethics committee (Ethics Committee of the Technical University of Munich, School of Medicine, Klinikum rechts der Isar; Approval number: 2022-317-S-SR) and was previously registered (https://clinicaltrials.gov/ct2/show/NCT05635552). All participants in this study provided written informed consent.

We recruited 43 patients from the PCS outpatient department (67.4%; 29/43) and through social media (32.6%, 14/43). Out of those, 41 patients (95.3%) were included in our study. One patient was excluded because they no longer exhibited PCS-typical symptoms during measurement, and one patient was excluded because there was no temporal association between SARS-CoV-2 infection and the onset of PCS symptoms. For patients recruited through social media, an initial survey was sent out with a questionnaire exploring acute SARS-CoV-2 infection and ongoing PCS-typical symptoms. Patients had to provide proof of a positive SARS-CoV-2 reverse transcription-polymerase chain reaction (RT-PCR) or rapid antibody test conducted at least 3 months ago, and they had to have a PCS-typical complaint complex ongoing for at least 2 months. Additionally, the temporal relationship between SARS-CoV-2 infection and the onset of PCS-typical symptoms and alternative diagnoses were reviewed. Exclusion criteria included missing or incomplete consent forms, age under 18 years, pregnancy, malignancy, diseases associated with a significant change in life expectancy, autoimmune diseases of the rheumatological type, cataract, epilepsy, and glaucoma. In cases of uncertainty, eligibility was discussed in a weekly meeting with the Chief Investigator (CI), Principal Investigator (PI), and the study base team, and decisions were made by majority vote. The healthy control (HC) group consisted of 204 participants recruited before the COVID-19 pandemic [31].

### Retinal vessel analysis

RVA was performed using the Dynamic Vessel Analyzer (DVA, commercial product DVAlight; IMEDOS Systems, Jena, Germany), and SVA measurements were performed using the Static Vessel Analyzer (IMEDOS Systems, Jena, Germany, based on TRC-NW8 non-mydriatic retinal camera; Topcon, Tokyo, Japan), as previously published [30]. Before the examination, pupils were dilated using topical tropicamide (0.5% Mydriaticum Stulln; Pharma Stulln, Germany), and patients were seated in a quiet dark room for a ten-minute rest period. Static analysis was performed before DVA.

For DVA, arteriole and venule segments of a length between 0.5 to 1 mm were analysed roughly two-disc diameters away from the optic nerve rim in the upper-temporal or lower temporal direction. Patients were instructed to focus on a needle attached inside the camera, and diameters of one arterial and one venous segment were automatically and continuously recorded for 350 s with the DVAlight device. The baseline recording was 50 s, followed by a flickering phase of 20 s, and then a recovery period of 80 s. Three cycles of these phases were performed in total. To ensure the highest quality standards and exclude substandard data, we compared the quality of the vessel response curves using a cumulative scoring method ranging from 0 to 5, as described earlier [30] Retinal images with a total score of < 2.5 were re-evaluated by a second experienced observer, and potentially excluded after reaching a consensus. In two patients, we were not able to obtain quality DVA data (score value < 2.5) due to a lack of information in the measured region and in one patient, DVA measurement had to be stopped due to excessive fatigue. For each individual patient and participant with quality data, the median arteriolar and venular vessel diameter of the three measurement cycles was calculated and plotted on a diameter-time curve (Graphical Abstract). We calculated the percentage of maximum dilation in relation to the baseline diameter (aFID and vFID), as described before [30].

For SVA analysis, at least three quality images from one eye were taken with a focus on the optic disc in the middle and at an angle of 50°. Two pictures with the highest quality were analysed using Vesselmap 2^®^ (IMEDOS Systems GmbH, Jena, Germany). Segments of retinal arterioles and veins were semi-automatically marked using a mask, within a ring 1 disc diameter away from the optic disc rim, and parameters of SVA were assessed using the Paar-Hubbard formula [32]. Diameters of arteries (CRAE) and veins (CRVE) were averaged with this formula, and the arteriolar-to-venular ratio (AVR) was calculated as the ratio between CRAE and CRVE. One measured unit of the imaging device relates to 1 µm in the model of Gullstrand's normal eye. If two independent analyses were performed by independent examiners, the mean value of CRAE, CRVE, and AVR was calculated. Previous studies have shown high reproducibility of the procedure [33]. Inter- and intra-observer inter-class correlation coefficients for CRAE and CRVE ranged from 0.75 to 0.87 [32, 34]. Thirty retinal images were re-analysed, and the correlation coefficients were 0.98 for CRAE, 0.97 for CRVE, and 0.97 for AVR, indicating a high reproducibility of static retinal vessel parameters [35].

### Clinical assessment

A standardized questionnaire assessed PCS symptoms, essential demographic characteristics, pre-existing illnesses, and medication on the recruitment day. Ongoing post-acute symptoms of the last two weeks were asked, with a focus on 12 symptom complexes, including chemosensory deficits, fatigue, exercise intolerance, joint or muscle pain, ear-nose-throat (ENT) ailments, coughing/wheezing, chest pain, gastrointestinal, neurological, and dermatological ailments, acute infection, and sleep disturbances. Reported symptoms were then encoded as 1 (persistent) and 0 (not constant), and the PCS severity score was calculated [36]. Patient-reported outcome measures (PROMs) focused on the assessment of fatigue (FSS) and depression (PHQ-9) [37, 38]. For patients with myalgic encephalomyelitis/chronic fatigue syndrome (ME/CFS), the Canadian Consensus Criteria for ME/CFS was used [39].

### Laboratory values

Blood sampling was performed as previously described [40]. Standard laboratory measurements were performed in an ISO-certified routine laboratory. IL-6, IL-8, CXCL10, MCP-1 and ICAM-1 in the patient`s serum were quantified using the Cytometric Bead Array Flex system (BD Biosciences, San Diego, US), according to the instructions of the manufacturer. For the determination of von Willebrand Factor (vWF) antigen (Ag) on human samples, the LIAPHEN^™^ vWF: Ag kit (Hyphen Biomed, Neuville-sur-Oise, France), an immunoturbidimetric assay, was used. The measurements were performed according to the manufacturer's instructions. Briefly, calibrator and controls were reconstituted as indicated in the specific instructions. The calibration concentrations were programmed from 0 to 150% vWF:Ag in Imidazole buffer. The specimens and controls were diluted 4:15 in the same buffer. A calibration curve was established and tested with the quality controls. For high concentrations, between 150 and 1600%, samples were pre-diluted in Imidazole buffer. The tests were performed at 37 °C, and the turbidity was measured at 575 nm.

### Statistical analysis

All statistical analyses were performed using R (Version: 4.2.1). The “All Eyes on PCS” study was designed following the Strengthening the Reporting of Observational studies in Epidemiology (STROBE) guidelines [41]. All statistical analysis were performed using R (Version: 4.2.1) and R Studio (Version: RStudio 2023.03.0 + 386). Normally distributed values are shown as mean ± standard deviation (SD), non-normally distributed as median and their inter-quartile-range (IQR) and categorical data are shown as counts and their percentages if not otherwise stated. To analyse distribution, data was visualized as boxplot and histogram and the Shapiro–Wilk test on normality was performed. To compare baseline characteristics Welch two-sample test was used for normally distributed values, Wilcoxon rank sum test for non-normal and χ^2^ – test for categorical value. We compared means of the DVA parameters aFID and vFID and SVA parameters CRAE, CRVE and AVR between PCS and matched healthy controls. For age- and gender matching we used the Matching package in R with exact matching for gender and a caliper for age. Success of matching was than controlled using the MatchBalance function. We fitted linear multivariate regression models to adjust for potential confounders [42–44]. Normality of residuals was assessed using the olsrr package. Histograms of residuals were inspected and the Shapiro–Wilk test on normality was used. All graphs were generated with ggplot2, for interaction blots we used the interaction package and for correlation plots we used the ggpubr package. The ggpubr package was also used to calculate Spearman’s correlation coefficient. To combine the two variables AVR and vFID we fitted a logistic regression model (Table E1: Online Supplement). The binary response variable was cohort dependency (PCS or healthy cohort) and two predictor variables were vFID and AVR. By fitting a regression model, we then estimated the relationship between the predictor variables and the probability of belonging to a particular cohort. The AUC was calculated for the respective regression model and the confidence interval was calculated using DeLong´s method. For assessment of discriminatory ability of biomarkers receiver operating characteristics analysis were performed with the plotROC and pROC package and areas under the curve (AUCs) are presented. To calculate p_interact_ we fitted a multivariate linear regression model with SVA parameters as the depended variables and the interaction terms PCS severity and level of inflammatory markers in PCS patients as two predictor variables (Table E2: Online Supplement). All values used for data analysis were typed in by two independent researchers in separate sheets and then checked for discrepancies (double-data verification). The graphical abstract was created with BioRender.com.

## Results

### Baseline characteristics

Forty-one patients (mean age 42.2 y ± 12.2, 75.6% female) with previous SARS-CoV-2 infection and exhibiting PCS-typical symptoms were recruited. Patients were age- and gender-matched with 41 healthy volunteers (41.8 y ± 13.7) from an HC group (*n* = 204). After matching, CV risk factors were not significantly different between the two cohorts. The most frequent comorbidities and chronic medication in the PCS cohort are shown in Table 1. During acute SARS-CoV-2 infection, four (9.8%) patients were hospitalized, and one (2.4%) patient was admitted to the intensive care unit (ICU). Patients who had been hospitalized with acute infection showed a tendency towards higher PCS severity scores (34.6 ± 9.3 vs. 45.6 ± 8.5, *p* = 0.07). There was no difference between virus variants in PCS severity scores (Fig. E1: Online Supplement). The median duration of PCS was ten months (7.0–18.0 months) with a mean PCS severity score of 35.7 (± 9.7). Fatigue (95.1%), exercise intolerance (90.2%), and brain fog (90.2%) were the three most abundant symptoms. Eight (19.5%) patients were laid off, and the median cumulative time off sick leave was 122.0 days (4.0–291.0 days). Standard laboratory parameters showed significantly higher leukocytes in PCS patients (Table 1).

**Table 1:** Baseline characteristics

Clinical characteristics	SARS-CoV-2 naïve (*n* = 41)	PCS (*n* = 41)	*P* value
Age			
Years, Mean (SD)	41.8 (± 13.7)	42.2 (± 12.2)	0.89
Gender			
Female	31 (75.6%)	31 (75.6%)	1.00
BMI			
kg/m^2^, Median (IQR)	23.6 (± 3.5)	24.2 (± 3.9)	0.55
RRsyst			
mmHg, Median (IQR)	120 (116–130)	118(112–130.5)	0.53
Cardiovascular risk factors			
Obesity	8 (25.8%)	15 (48.4%)	0.60
Nicotine abuse	5 (16.1%)	6 (19.4%)	1.00
Art. hypertension	7 (22.6%)	8 (25.8%)	1.00
DM	0 (0.0%)	0 (0.0%)	1.00
Hypercholesterolemia	–	19 (61.3%)	-
Acute SARS-CoV-2 Infection			
Severity of acute infection			
0	–	0 (0.0%)	–
2	–	24 (58.5%)	–
3	–	13 (31.7%)	-
4	–	2 (4.9%)	–
5	–	1 (2.4%)	–
6	–	1 (2.4%)	–
Variance	–		
Alpha	–	3 (7.3%)	–
Delta	–	7 (17.1%)	–
Omicron	–	10 (24.4%)	–
Unknown	-	21 (51.2%)	–
Number of vaccinations			
0	–	3 (7.3%)	–
2	–	15 (36.6%)	–
–	-	23 (56.1%)	–
PCS characteristics			
PCS duration			
months, Median (IQR)	–	10.0 (7.0—18.0)	–
PCS severity score			
Mean (SD)	–	35.7 (9.73)	–
Fatigue	–	39 (95.1%)	–
Exercise intolerance	–	37 (90.2%)	–
Brain fog	–	37 (90.2%)	–
Cumulative days of sick leave			
days, Median (IQR)	–	122.0 (4.0—291.0)	–
Work-loss due to PCS	–	8 (19.5%)	–
Comorbidities			
Hypothyroidism	–	8 (19.5%)	–
Bronchial asthma	–	6 (14.6%)	–
Medication			
ACE2-inhibitors	–	3 (7.3%)	–
ß-blockers	–	5 (12.2%)	–
Psychiatric/sleeping medication	–	8 (19.5%)	–
L-Thyroxin	–	8 (19.5%)	–
Asthma inhaler	–	6 (14.6%)	–
Lab parameters			
Leukocytes			
G/L, Mean (SD)	5.3 (4.7–6.3)	6.2 (5.3–7.4)	0.027*
Hb			
mg/dl, Median (IQR)	14.9(13.7 – 15.3)	14.1 (13.5–14.4)	0.095

*P*-values are shown for statistical tests comparing PCS patients (*n* = 41) with SARS-CoV-2 infection naive participants (*n* = 41); t-test was used for normally distributed variables, the χ^2^ test for categorical variables, the Wilcoxon rank sum test for variables with a skewed distribution, and Fisher’s exact test for binary variables. BMI, body mass index; RRsyst, systolic blood pressure; Art., arterial; DM, diabetes mellitus I or II; obesity is defined as BMI > 25 kg/m^2^; hypercholesterolemia is defined as cholesterol > 200 mg/dl. Severity of acute infection was assessed using the WHO clinical progression scale [44]. PCS severity score was assessed using the score published by Bahmer et al. [35]

### Retinal vessel reactivity to flickering light (DVA) and structural aspects of retinal microvasculature (SVA) in PCS

**Fig. 1:**
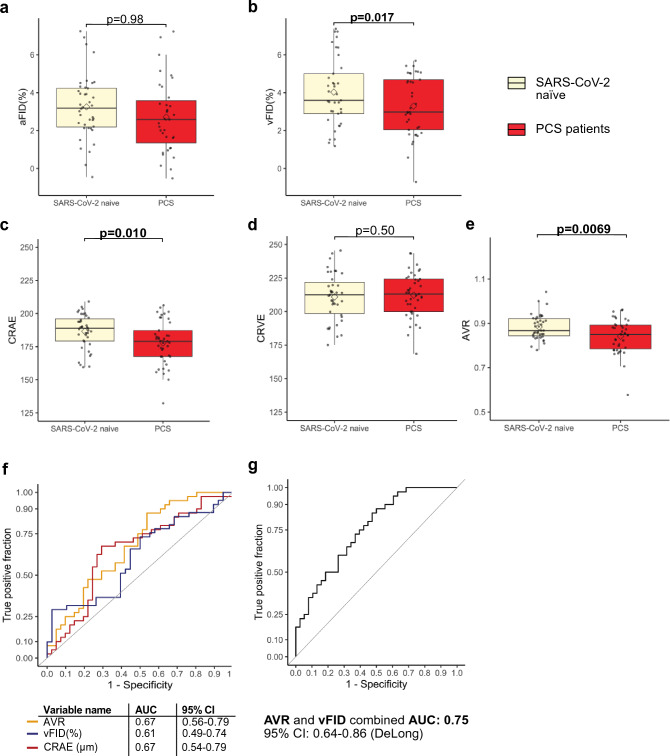
Flicker induced dilation and static retinal vessel analysis in PCS patients and HC control.** a **Boxplots show arteriolar dilatation (aFID) and venular dilation (vFID) (**a **and **b)** and SVA parameters CRAE, CRVE and AVR (**c**, **d **and **e**) in PCS patients (red) and SARS-CoV-2 naïve participants (beige). For RVA parameters boxplots represents data of *n* = 39 PCS patients and *n* = 39–40 SARS-CoV-2 naïve participants and for SVA parameters data of *n* = 41 PCS patients and n= 40 SARS-CoV-2 naïve participants. Mean values are shown as a rectangle and median values as a line. Wilcoxon rank sum test (skewed distributions) or Welch´s t test (normal distribution) were used to compare groups. **f **ROC curves show discrimination between PCS patients and infection naïve participants for AVR, vFID and CRAE. **g** ROC curve shows discrimination between PCS patients and infection naïve participants for AVR and vFID combined. AUC is presented with 95% confidence intervals (CI) calculated with DeLong´s method.

After matching, PCS patients showed significantly lower venular dilation after stimulation when compared to HC (vFID; 3.42% ± 1.77% vs. 4.64% ± 2.59%; *p* = 0.02), suggesting a persistent alteration of microvascular endothelial function in PCS. There was no difference in arteriolar dilation (aFID) between the two cohorts (aFID; 3.26% ± 2.71% vs. 3.25% ± 1.69%; *p* = 0.98) (Fig. 1 a and b).

To assess retinal microvasculature in PCS patients, retinal fundus pictures were analyzed. PCS patients showed significantly narrower retinal arterioles, indicated by lower CRAE when compared with HC (178.1 [167.5–190.2] vs. 189.1 [179.4–197.2], *p* = 0.01). We did not observe differences in the central retinal venular equivalent (CRVE) between cohorts (213.1 [200.0–224.2] vs.212.2 [197.1–220.8], *p* = 0.5). Subsequently, the arteriolar venular ratio (AVR) was significantly lower in PCS patients as it is the ratio of CRAE/CRVE (0.84 [0.8–0.9] vs. 0.88 [0.8–0.9], *p* = 0.007) (Fig. 1 c, d and e). After controlling for potential confounders, lower venular dilation (*p* = 0.03) and lower AVR (*p* = 0.048) remained associated with PCS. The strength of association with narrower retinal arterioles was smaller after adjustment (*p* = 0.077) (Table E3 Online Supplement).

To test whether RVA parameters could serve as a biomarker for PCS, we plotted receiver operated characteristics (ROC) curves and calculated the area under the curve (AUC) of variables. RVA parameters showed acceptable discrimination of the two cohorts for vFID (AUC: 0.61), CRAE (AUC: 0.67) and AVR (AUC: 0.67). When combining both parameters AVR and vFID in a logistic regression model the corresponding score reached good discrimination between HC and PCS patients (AUC: 0.75) (Fig. 1 f and g).

### PCS severity amplifies changes of retinal microcirculation

In this PCS cohort, 34 (82.9%) patients were severely affected (PCS severity score > 26.25), and 7 (17.1%) patients were moderately affected (PCS severity score > 10.75 and ≤ 26.25). Severely affected patients were more obese (0.0% vs. 44.1%; *p* = 0.04) and as expected, Fatigue Severity Scale (FSS) was higher (4.1 [3.6–5.8] vs. 6.1 [5.8–6.7], *p* = 0.02). Severely affected patients showed tendencies towards more PCS-related work loss (0% vs. 23.5%, *p* = 0.3) and cumulative days of sick leave (106.0 [0.0–157.0] vs. 122.5 [15.5–303.8], *p* = 0.2). Laboratory parameters showed no relevant differences between groups (Table E4: Online Supplement).

**Fig. 2:**
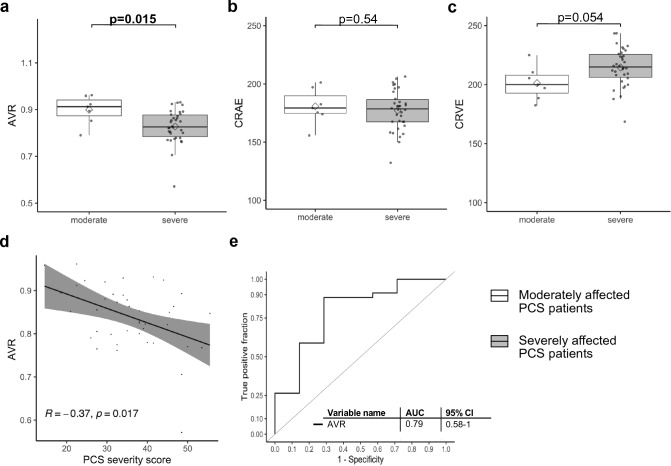
Parameters of retinal microvasculature in severely affected PCS patients.** a**, **b** and **c **Boxplot of SVA parameters CRAE, CRVE and AVR in seven moderately affected PCS patients (PCS severity score > 10.75 and ≤26.25) (white) and 34 severely affected PCS patients (PCS severity score >26.25,) (grey). Boxplots show values as mean (rectangle) and median (line). To compare groups Wilcoxon rank sum test was used for skewed data and Welch´s t-test for normally distributed data. **d **Scatterplot shows association between PCS severity score and AVR and the corresponding R and p-value. **e** ROC curves show discrimination between moderately and severely affected patients for AVR and the AUC with 95% confidence intervals (CI) calculated with DeLongs test.

For DVA parameters, we did not see any relevant differences between moderately and severely affected patients (Fig. E2: Online Supplement). For SVA parameters, we observed a trend towards narrower retinal arterioles (CRAE) and wider retinal venules (CRVE), both failing to reach significance (Fig. 2 b and c). AVR was significantly lower in severely affected PCS patients (0.83 [0.78–0.88] vs. 0.91 [0.87–0.94]; *p* = 0.02) and proved to be a good marker to discriminate between severely and moderately affected PCS patients (AUC:0.79) (Fig. 2 a and e). The combination of AVR and vFID did not reach a better discriminatory ability then solely AVR (not shown). Spearman’s correlation coefficient shows a negative, correlation between AVR and PCS Score (*R* = − 0.37 *p* = 0.017) (Fig. 2 d) and the association of lower AVR with higher PCS score (*p* = 0.002) and severe phenotype (*p* = 0.03) remained significant after adjusting for potential confounders (Table 2).

**Table 2:** Association of AVR with PCS severity. Arteriolar venular ratio (AVR) as the dependent variable

Characteristics	Univariate		Multivariate^b^		
	β-Coefficient	p-value	β-Coefficient	p-value	R^2^/ R^2^ adjust
Age, year	− 0.0007	0.71	–	–	
Gender, male	− 0.04	0.10	–	–	
Obesitiy	− 0.05	0.012*	–	–	
Art. hypertension	− 0.009	0.75	–	–	
Nicotine abuse	− 0.02	0.62	–	–	
PCS score	− 0.003	0.0043**	− 0.004	0.0015**	**0.46/0.36**
Severe phenotype	− 0.071	0.019*	− 0.070	0.027*	**0.37/0.26**

#### Chronic fatigue in PCS and impairment of retinal microcirculation

Chronic fatigue is one of the most debilitating symptoms in PCS patients, and SARS-CoV-2 infection has been reported to cause ME/CFS [46]. We evaluated whether there is an association between RVA and ME/CFS.

In our cohort, 60.9% (25/41) of PCS patients met the Canadian Consensus Criteria for ME/CFS. PCS patients with CFS were more obese (12.5% vs. 52.0%, *p* = 0.02); other CV factors were not different. PCS patients with CFS had a higher PCS severity score (30.4 ± 8.7 vs. 39.1 ± 8.9, *p* = 0.004) and were more fatigued and depressed, indicated by higher FSS (5.8 [4.1–6.2] vs. 6.2 [5.8–6.9], *p* = 0.02) and higher PHQ9 score (8.9 ± 4.6 vs. 11.8 ± 4.1, *p* = 0.040). There were no relevant differences in laboratory values between the two groups (Table E5: Online Supplement).

**Fig. 3:**
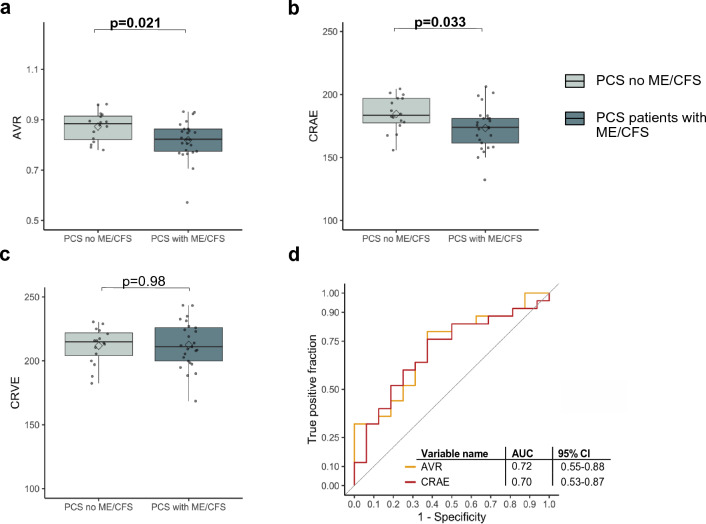
Comparison of SVA parameters in PCS patients with and without CFS.** a**, **b** and c Boxplot of SVA parameters CRAE (µm), CRVE (µm), and AVR in PCS patients without ME/CFS (blue, *n *= 16) and PCS patients with ME/CFS (grey, *n* = 25). Boxplots show values as mean (rectangle) and median (line). To compare groups Wilcoxon rank sum test was used for skewed data. **d** ROC curves show discrimination between ME/CFS and none ME/CFS for AVR and CRAE and their AUC with 95% confidence intervals (CI), calculated with DeLong’s test.

We observed significantly narrower retinal arterioles in PCS patients with CFS, indicated by lower CRAE (183.5 [177.4–197.0] vs. 174.0 [161.5–181.0], p = 0.03). There was no difference in the size of retinal venules between groups (214.9 [204.1–221.9] vs. 211.1 [199.9–226.0], *p* = 0.98) (Fig. 3 b and c). AVR was significantly lower in PCS patients with CFS (0.88 [0.82–0.91] vs. 0.82 [0.77–0.86], *p* = 0.02), and both AVR (AUC: 0.72) and CRAE (AUC: 0.70) were good markers to distinguish between PCS patients with or without CFS (Fig. 3 a and d).

After controlling for confounders of SVA, association for lower AVR (p = 0.3) and narrower retinal arterioles (*p* = 0.04) with CFS was smaller (Table E6: Online Supplement).

### Association of chronic inflammation and microvascular alterations in PCS

Higher levels of CXCL10 (*p* = 0.01), D-Dimer (*p* = 0.03), and TAG (*p* = 0.03) were significantly associated with lower AVR (Table 3 a). Narrower retinal arterioles (CRAE) showed no associations with inflammatory parameters, however, with higher levels of D-Dimer (*p* = 0.008) and TAG (*p* = 0.002) (Table 3 b). Wider retinal venules were significantly associated with higher levels of CXCL10 (*p* = 0.02) and IL-6 (*p* = 0.04) (Table 3 c). Biomarkers of ED such as von Willebrand factor (vWF) and ICAM-1 showed no significant associations with SVA parameters. However, we did observe a modest association between lower AVR and increased vWF-activity (Table 3 a). Subsequently, we compared AVR values between PCS patients with increased vWF-activity (> 200%), reduced vWF-activity (< 50%) and normal vWF-activity (50–200%) [46]. AVR was significantly lower in patients with increased vWF-activity when compared with normal range (0.70 ± 0.18 vs. 0.85 ± 0.06, *p*_Holm_ = 0.014) or reduced vWF-activity (0.70 ± 0.18 vs. 0.85 ± 0.08, p_Holm_ = 0.030) (Fig. E3: Online Supplement). Increased vWF-activity was associated with higher PCS-severity scores in patients (Table E7: Online Supplement).

**Table 3:** Associations of laboratory parameters with variables of microcirculation

a. Arteriolar venular ratio (AVR) as the dependent variable			
Characteristics	Multivariate^b^		R^2^/R^2^ adjusted
	β-Coefficient	*P*-value	
**D Dimer (ug/I)**	− 0.0002	0.028*	0.37/0.23
Thrombocytes (G/l)	0.0003	0.26	0.27/ 0.12
vWF (%)	− 0.0005	0.09	0.33/0.21
**TAG (mg/dl)**	− 0.0005	0.031*	0.36 / 0.23
Cholesterol(mg/dl)	0.0003	0.33	0.28/0.14
Ferritin (mg/dl)	− 0.0003	0.083	0.34/0.21
**CXCL10 (pg/ml)**	− 0.001	0.014*	0.38/ 0.26
IL-8 (fg/ml)	− 0.000003	0.82	0.25/0.11
IL-6 (pg/ml)	− 0.001	0.36	0.29/0.16
MCP-1 (pg/ml)	− 0.0001	0.68	0.25/0.11
CRP (mg/dl)	0.016	0.64	0.26/0.11
ICAM-1(pg/ml)	− 0.0000006	0.96	0.27/0.14
Leukocytes (G/l)	0.006	0.43	0.25/0.10
Neutrophils (%)	0.0004	0.75	0.24/0.08
Lymphocytes (%)	0.0003	0.78	0.24/0.09
Hb (g/dI)	− 0.009	0.61	0.24/0.09
IgG (mg/dl)	− 0.00006	0.45	0.27/0.10
IgG4 (mg/dl)	0.0003	0.32	0.29/0.15

b. Central retinal arteriolar equivalent (CRAE) as the dependent variable			
Characteristics	Multivariate^b^		
	*β*-Coefficient	*P*-value	R^2^/R^2^ adjusted
**D Dimer (ug/I)**	− 0.05	0.008**	0.34 /0.20
Thrombocytes(G/I)	0.05	0.33	0.26 0.12
vWF(%)	− 0.05	0.36	0.20/0.05
**TAG (mg/dl)**	− 0.13	0.004**	0.42/ 0.31
Cholesterol (mg/dl)	0.014	0.80	0.24/0.09
Ferritin (mg/dl)	− 0.08	0.069	0.28/0.14
CXCL10 (pg/ml)	− 0.06	0.63	0.18/ 0.02
IL-8 (fg/ml)	− 0.004	0.22	0.21/0.06
IL-6 (pg/ml)	0.2	0.44	0.18/ 0.03
MCP-1 (pg/ml)	− 0.07	0.35	0.20/0.04
CRP (mg/dl)	8.4	0.25	0.27/ 0.13
ICAM-1 (pg/ml)	− 0.001	0.66	0.18/032
Leukocytes (G/I)	1.44	0.35	0.26/0.11
Neutrophils (%)	0.17	0.61	0.23/0.07
Lymphocytes (%)	− 0.008	0.97	0.24/0.08
Hb(g/dI)	− 3.02	0.43	026/0.11
IgG (mg/dl)	0.0002	0.99	0.22/0.07
**IgG4 (mg/dl)**	0.16	0.047^a^	0.32/0.19

c. Central retinal venular equivalent (CRVE) as the dependent variable			
Characteristics	Multivariate^b^		R^2^/R^2^ adjusted
	β-Coefficient	*P-value*	
D Dimer (ug/I)	− 0.01	0.40	0.18/0.007
Thrombocytes(G/I)	0.01	0.77	0.35 0.22
vWF(%)	− 0.05	0.39	0.25/0.11
TAG (mg/dl)	− 0.04	0.34	0.35/0.22
Cholesterol (mg/dl)	− 0.05	0.31	0.35/0.22
Ferritin (mg/dl)	− 0.07	0.087	0.20/0.03
**CXCL10 (pg/ml)**	0.3	0.020*	0.35/0.22
**I**L-8 (fg/m**l**)	− 0.006	0.054	0.31/0.17
**IL-6 (pg/ml)**	0.6	0.040*	0.32/0.20
IgG4 (mg/d**l**)	0.09	0.25	0.34/0.21
MCP-1(pg/ml)	− 0.05	0.49	0.23/0.08
CRP (mg/dl)	5.9	0.35	0.35/0.22
ICAM-1 (pg/ml)	− 0.001	0.62	0.23/0.10
Leukocytes (G/I)	0.26	0.84	0.35/0.21
Neutrophils (%)	0.13	0.63	0.36/0.22
Lymphocytes (%)	− 0.17	0.51	0.35/0.23
Hb(g/dI)	− 1.5	0.64	0.34/0.22
IgG (mg/dl)	0.015	0.37	0.33/0.20
IgG4 (mg/dl)	0.9	0.25	0.34/0.21

The multivariate linear regression model shows associations of laboratory parameters with the dependent variables AVR, CRAE, CRVE. D-Dimer was measured in *n* = 36, thrombocytes, leukocytes neutrophils, lymphocytes, hemoglobin (Hb) and ferritin in *n* = 37, CRP (C-reactive protein), Cholesterol, TAG, CXCL10, IL8, IL-6, IgG, IgG4 and MCP-1 in *n* = 38. Von Willebrand factor (vWF) and ICAM-1 in *n* = 40. In the multivariate modelb, age, gender, obesity, arterial hypertension and nicotine abuse were included. The respective R2 are shown for the multivariate modelb after correction. For model^a^ F-statistic was not significant

As we observed associations of inflammatory biomarkers with microcirculation parameters, we were interested, whether chronic inflammation would amplify our observed correlation between microvascular changes and PCS severity score. Therefore, we fitted a regression model with interaction effects between PCS severity score and laboratory parameters.

**Fig. 4:**
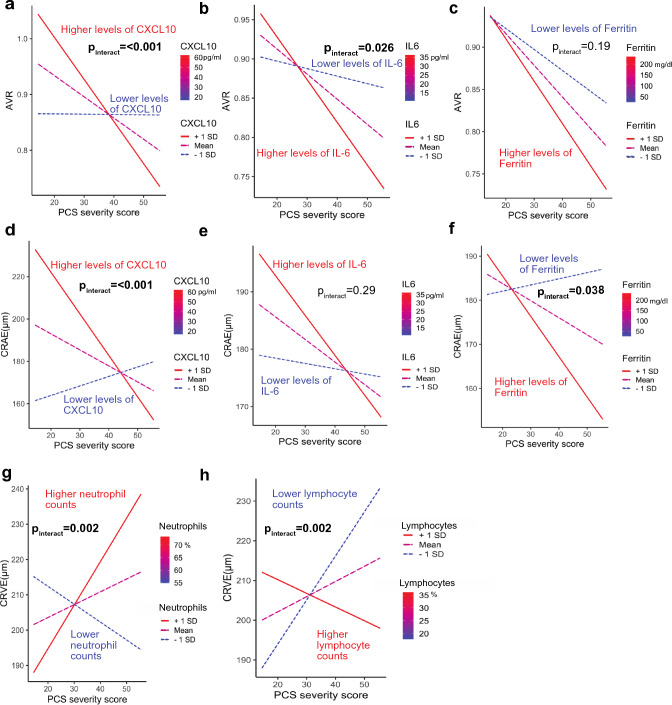
Interactions between chronic inflammatory parameters, severe PCS and endothelial dysfunction. Interaction plots show the association between AVR (**a**, **b** and **c**), CRAE (**d**, **e**, and **f**); and CRVE (**g** and **h**) and PCS score for three different levels of inflammatory variables selected in the range of the observed data. Regression lines are plotted as “higher levels of the variable” (+ 1 standard deviation, red line), Mean of the variable (purple dashed line) and “Lower levels of the variable” (− 1 standard deviation, blue dotted line). Lymphocyte counts and neutrophil counts were measured in *n* = 37 and *n* = 35 patients. *P*_interact_ values indicate significant interactions between PCS severity score and the inflammatory variable and was computed using a multivariate linear regression model controlled for confounders age, gender, obesity, arterial hypertension and nicotine abuse.

The described association between lower AVR and higher PCS severity scores was stronger in patients exhibiting higher levels of CXCL10 or IL-6. We observed interactions between CXCL10 and PCS-severity score (*p*_interact_ < 0.001) and interactions between IL-6 and PCS severity score (*p*_interact_ = 0.03) (Fig. 4 a and b). vWF activity did not show significant interactions with PCS severity and AVR (Fig. E3: Online Supplement). In line with this, the association between high PCS severity score and lower CRAE was more pronounced in PCS patients with higher CXCL10 levels (*p*_interact_ < 0.001) and higher ferritin levels (p_interact_ = 0.04) (Fig. 4 d and f).

The association between higher CRVE and lower PCS severity was not influenced by CXCL10 and IL-6 (Fig. E4: Online Supplement), however by higher neutrophil counts (*p*_interact_ = 0.002) and lower lymphocyte counts (*p*_interact_ = 0.002) (Fig. 4 g and h).

## Discussion

This observational prospective cohort study is the first to show prolonged endothelial dysfunction using SVA and DVA in PCS patients. We observed significantly lower venular dilation (vFID), narrower CRAE, and lower AVR in PCS patients. We showed that a combination of DVA and SVA parameters could be a valuable biomarker for diagnosing PCS if validated in independent studies. Notably, PCS severity was associated with more pronounced microvascular alterations in SVA.

Acute SARS-CoV-2 infection leads to microvascular impairment, which is best described by the presence of endotheliitis and loss of vascular integrity [11, 16]. One study reported changes in retinal vessel diameters in acute SARS-CoV-2 infected patients, which resolved in non-severely affected patients after six months [48]. We could now show that PCS patients cannot restore primary damage to the endothelium. Since retinal vessel integrity is a surrogate of pan-endothelial health, persistent ED could partly answer persisting symptoms in PCS.

DVA and SVA can assess retinal structure and function by differentiating between arteries and veins. We observed reduced flicker-induced dilation in venules, however, not in arterioles. This aligns with our previous cohort studies where only venular, not arteriolar dilation, proved to be a promising biomarker for predicting all-cause mortality in hemodialysis patients and was independently associated with IL-6 [30]. It has been speculated that ongoing inflammation contributes to the observed alterations of ED in PCS. However, after correcting for confounders, we did not find associations between inflammatory parameters and vFID. This might be attributed to the smaller sample size and lower inflammatory levels in PCS patients compared to dialysis patients.

Concerning retinal vessel diameters, we found narrower arterioles. This is reflected in lower AVR, which proved the most reliable parameter in distinguishing PCS from HC and showed the strongest association with PCS severity. While CRAE and CRVE both display particular and separate microvascular patterns of the arterial and venous circulation, the AVR provides a non-specific measurement of the general regulatory state of retinal microcirculation [23]. Calibers of CRAE and CRVE are associated with CV risk factors such as nicotine abuse, age, diabetes, obesity, and higher blood pressure [23, 24, 42, 49]. Although the observed associations between PCS, PCS severity and AVR could be partly explained by the influence of confounding factors, analyses suggest that the effects of SARS-CoV-2 infection on microvascular integrity and endothelial function are likely to be prolonged and contribute to long-term consequences of the infection, rather than simply being a result of pre-existing cardiovascular morbidity. With a median PCS duration of 10 months in our cohort, this is a highly relevant clinical finding, especially since the severity of acute infection was mostly mild to moderate.

The ease of use of RVA could provide a deeper insight into endothelial health in PCS patients in clinical routine and may answer the call for endothelial biomarkers in COVID-19 proposed by the consensus paper of the ESC [50].

PCS patients with CFS exhibited a more severely impaired retinal microvascular integrity in our cohort. Even though this association was weaker after controlling for confounders, this aligns with recent findings describing ED in PCS patients with additional CFS [46] and patients with CFS independently of SARS-CoV-2 infection [51, 52]. Besides SARS-CoV-2, many viruses are known to infect endothelial cells and trigger endotheliitis, and post-viral fatigue is causally attributed more and more to ED [53].

The mechanisms that underlie ongoing inflammation in PCS patients are not fully clear. Autoimmunity, the persistence of the virus, and virus reactivation in patients have been discussed and could explain persistent ED in PCS patients [54–57]. Induction of autoimmunity after viral infections is not specific to SARS-CoV-2 and has been described in Epstein-Barr virus (EBV), cytomegalovirus, and many more [58].

We showed that higher levels of CXCL10 and IL-6 both amplify the observed association between PCS severity and lower CRAE and AVR. CXCL10 binds to CXCR-A expressed by leukocytes and the isoform CXCR3-B expressed by epithelial and endothelial cells. Binding to CXCR-B inhibits cell migration and stimulates cell apoptosis [59]. CXCL10 has been associated with alterations of neuroendocrine regulation and alterations in cognitive function [60]. Thus, potentially explaining the stronger phenotype of severe PCS and affected microcirculation in patients with higher CXCL10 levels. IL-6 has been shown to play a significant role in the pathogenesis of neurodegenerative diseases and has been linked to cognitive impairment in Alzheimer´s disease [61]. IL-6, as a driver of neuroinflammation, has been associated with ongoing neurocognitive and neuropsychiatric symptoms in patients after SARS-CoV-2 infection [14, 62]. The connection between the retinal microvasculature and the brain lies in the concept of the "retina-brain axis". Future studies could benchmark PCS symptoms, measure cerebral blood flow using MRI, and correlate these with parameters of the retinal microvasculature. This integrated approach holds promise for shedding light on the connection between retinal health and brain function. In case of wider CRVE we found a significant interaction between PCS severity and higher neutrophil and lower lymphocyte counts. NET formation and neutrophil-secreted inflammatory parameters are associated with post-COVID-19 pulmonary sequalae and patients with persistent lymphopenia have been shown to experience more post-acute symptoms [63, 64]. Further studies are needed to clarify the exact relationship between ED and chronic inflammation in PCS and the molecular causes of an unregulated immune response.

Neuropsychiatric symptoms are common in PCS, and due to the absence of objective markers for diagnosis, the assessment and quantification of persistent symptoms in patients can be challenging for physicians. This leads to a high level of frustration in patients and treating physicians. Here we show that ongoing symptoms in patients can be objectified using RVA and that quantifying microvascular endothelial function could improve the clinical management of PCS patients. High hopes lie in the development of therapy for PCS. In a preliminary preprint study, Bramante et al. demonstrated a protective effect of metformin in the development of PCS [65]. Metformin improves endothelial function via increased NO production, preventing endothelial cell injury and reducing leukocyte adhesion [66]. Flicker-induced dilation in retinal vessels is mainly a direct consequence of NO release due to neurovascular coupling [23]. In conclusion, RVA might be a valuable tool in PCS patients for diagnosis and therapy monitoring.

There are several limitations we must address. This is a cross-sectional, single-center cohort study, and the associations – as plausible as they are – should not be mistaken for causality. Although measurement of ED in PCS patients may have additive value in clinical management, the findings are exploratory, hypothesis-generating, and need to be validated in larger, independent cohorts. Furthermore, one limitation is the lack of a control group of participants who completely recovered after acute SARS-CoV-2 infection. Most of our patients were recruited in the PCS outpatient department of the LMU Munich. This ensured a correct diagnosis of PCS, but led to increased recruitment of patients, which were severely affected. This could diminish the significance and is a selection bias, as it is not clear whether the findings are also transferable to less severely affected patients. Although we have noted a correlation between inflammatory parameters and alterations in retinal microcirculation, we have yet to fully elucidate the precise mechanism by which SARS-CoV-2 infection contributes to prolonged endothelial dysfunction (ED). To gain a comprehensive understanding of the underlying biological pathophysiology, it will be important to incorporate a basic science approach that is currently lacking in our study.

## Conclusion and outlook

Taken together in this cohort of patients with PCS, symptoms were associated with persistent impaired microvascular integrity, amplified by higher levels of pro-inflammatory cytokines CXCL10 and IL-6. As RVA parameters are a surrogate of pan-endothelial health, this might explain the pathophysiological background of PCS. ED is increasingly studied as an independent risk factor in post-viral diseases and autoimmune conditions, leading to excessive fatigue and cognitive disorders. Our results highlight the importance of further investigating retinal vessel diameters and flicker light-induced dilation as potential biomarkers for these conditions, which could inform the development of new diagnostic and treatment strategies for patients suffering from PCS and related disorders.

## Supplementary Information

Below is the link to the electronic supplementary material.

Supplementary file1 (DOCX 18703 kb)

## Data Availability

Data are available from the corresponding author upon reasonable request.

## Abbreviations

Ag: Antigen
ARIC: The Atherosclerosis Risk In Communities
AUC: Areas under the curve
AVR: Arteriolar-venular ratio
CI: Chief Investigator
CRAE: Central retinal artery equivalent
CRP: C-reactive protein
CRVE: Central retinal vein equivalent
CV: Cardiovascular
CXCL10: C-X-C motif chemokine ligand 10
DVA: Dynamic vessel analysis
ED: Endothelial dysfunction
ENT: Ear-nose-throat
ESC: European Society of Cardiology
FMD: Flow-mediated dilation
FSS: Fatigue severity scale
Hb: Hemoglobin
HC: Healthy control
ICAM-1: Intercellular adhesion molecule 1
IL-6: Interleukin-6
ICU: Intensive care unit
IQR: Inter-quartile-range
ME/CFS: Myalgic encephalomyelitis/chronic fatigue syndrome
NO: Nitric oxide
OCT: Optical coherence tomography
PAT: Peripheral arterial tone
PHQ-9: Patient Health Questionnaire 9
PROM: Patient reported-outcome measures
PI: Principal Investigator
PCS: Post-COVID-19 syndrome
ROC: Receiver Operating Characteristic
RT-PCR: Reverse transcription- polymerase chain reaction
RVA: Retinal Vessel Analysis
SD: Standard deviation
STROBE: Strengthening the Reporting of Observational studies in Epidemiology
SVA: Static retinal vessel analysis
vWF: Von-Willebrand factor
