# Correction: Persistent endothelial dysfunction in post-COVID-19 syndrome and its associations with symptom severity and chronic inflammation

**DOI:** 10.1007/s10456-023-09892-7

**Published:** 2023-08-23

**Authors:** Timon Kuchler, Roman Günthner, Andrea Ribeiro, Renate Hausinger, Lukas Streese, Anna Wöhnl, Veronika Kesseler, Johanna Negele, Tarek Assali, Javier Carbajo-Lozoya, Maciej Lech, Heike Schneider, Kristina Adorjan, Hans Christian Stubbe, Henner Hanssen, Konstantin Kotliar, Bernhard Haller, Uwe Heemann, Christoph Schmaderer

**Affiliations:** 1 School of Medicine, Klinikum Rechts Der Isar, Department of Nephrology, Technical University of Munich, Ismaninger Str. 22, 81675 Munich, Germany; 2 Faculty of Health Care, Niederrhein University of Applied Sciences, Krefeld, Germany; 3 Medizinische Klinik Und Poliklinik IV, LMU University Hospital Munich, Ziemssenstraße 5, 80336 Munich, Germany; 4 School of Medicine, Klinikum Rechts Der Isar, Department of Clinical Chemistry and Pathobiochemistry, Technical University of Munich, Ismaninger Str. 22, 81675 Munich, Germany; 5 Department of Psychiatry and Psychotherapy, LMU University Hospital Munich, Nußbaumstraße 7, 80336 Munich, Germany; 6 Medizinische Klinik Und Poliklinik II, LMU University Hospital Munich, Marchioninistraße 15, 81377 Munich, Germany; 7 Department of Sport, Exercise and Health, Preventive Sports Medicine and Systems Physiology, University of Basel, Basel, Switzerland; 8 Aachen University of Applied Sciences, Heinrich-Mussmann-Str. 1, 52428 Jülich, Germany; 9 School of Medicine, Institute for AI and Informatics in Medicine, Technical University of Munich, Klinikum Rechts Der Isar, Ismaninger Str. 22, 81675 Munich, Germany; 10 German Centre for Infection Research (DZIF), Partner Site Munich, Munich, Germany

**Correction to: Angiogenesis** 10.1007/s10456-023-09885-6

In the original publication of the article, the author wishes to update the funding section. The updated funding information is provided below.

The original article has been corrected.

