# Correction: Persistent endothelial dysfunction in post-COVID-19 syndrome and its associations with symptom severity and chronic inflammation

**DOI:** 10.1007/s10456-025-10007-7

**Published:** 2025-10-10

**Authors:** Timon Kuchler, Roman Günthner, Andrea Ribeiro, Renate Hausinger, Lukas Streese, Anna Wöhnl, Veronika Kesseler, Johanna Negele, Tarek Assali, Javier Carbajo‑Lozoya, Maciej Lech, Heike Schneider, Kristina Adorjan, Hans Christian Stubbe, Henner Hanssen, Konstantin Kotliar, Bernhard Haller, Uwe Heemann, Christoph Schmaderer

**Affiliations:** 1 School of Medicine, Klinikum Rechts Der Isar, Department of Nephrology, Technical University of Munich, Ismaninger Str. 22, 81675 Munich, Germany; 2 Faculty of Health Care, Niederrhein University of Applied Sciences, Krefeld, Germany; 3 Medizinische Klinik Und Poliklinik IV, LMU University Hospital Munich, Ziemssenstrase 5, 80336 Munich, Germany; 4 School of Medicine, Klinikum Rechts Der Isar, Department of Clinical Chemistry and Pathobiochemistry, Technical University of Munich, Ismaninger Str. 22, 81675 Munich, Germany; 5 Department of Psychiatry and Psychotherapy, LMU University Hospital Munich, Nusbaumstrase 7, 80336 Munich, Germany; 6 Medizinische Klinik Und Poliklinik II, LMU University Hospital Munich, Marchioninistrase 15, 81377 Munich, Germany; 7 Department of Sport, Exercise and Health, Preventive Sports Medicine and Systems Physiology, University of Basel, Basel, Switzerland; 8 Aachen University of Applied Sciences, Heinrich‑Mussmann‑Str. 1, 52428 Julich, Germany; 9 School of Medicine, Institute for AI and Informatics in Medicine, Technical University of Munich, Klinikum Rechts Der Isar, Ismaninger Str. 22, 81675 Munich, Germany; 10 German Centre for Infection Research (DZIF), Partner Site Munich, Munich, Germany

**Correction to: Angiogenesis (2023) 26:547–563**

10.1007/s10456-023-09885-6

In the original publication of the article, the author wishes to update the funding section. The updated funding information is provided below. Also the author’s name Konstantin Kotliar was incorrectly written as Konstantin Kotilar.

The original article has been corrected.

