# Supplementary material for: Persistent endothelial dysfunction in post-COVID-19 syndrome and its associations with symptom severity and chronic inflammation
Source: Angiogenesis. 2023 Jul 28;26(4):547–63. doi: 10.1007/s10456-023-09885-6 (PMC10542303; doi:10.1007/s10456-023-09885-6)
Supplement: Supplementary file 1 — Supplementary file1 (DOCX 18703 kb) [file 10456_2023_9885_MOESM1_ESM.docx]

**Persistent endothelial dysfunction in post-COVID-19 syndrome and its associations with symptom severity and chronic inflammation**

Timon Kuchler^1^; Roman Günthner^1^; Andrea Ribeiro^1,3^; Renate Hausinger^1^; Lukas Streese^2^; Anna Wöhnl^1^; Veronika Kesseler^1^; Johanna Negele^1^; Tarek Assali^1^; Javier Carbajo-Lozoya^1^; Maciej Lech^3^; Heike Schneider^4^; Kristina Adorjan^5^; Hans Christian Stubbe^6^; Henner Hanssen^7^; Konstantin Kotilar^8^; Bernhard Haller^9^; Uwe Heemann^1^; Christoph Schmaderer^1,10^

Authors are affiliated with: ^1^ “Technical University of Munich, School of Medicine, Klinikum rechts der Isar, Department of Nephrology, Ismaninger Str. 22, 81675 Munich, Germany; ^2^ Faculty of Health Care, Niederrhein University of Applied Sciences, Krefeld, Germany. ^3^ LMU University Hospital Munich, Medizinische Klinik und Poliklinik IV, Ziemssenstraße 5, 80336 Munich, Germany“; ^4^“Technical University of Munich, School of Medicine, Klinikum rechts der Isar, Department of Clinical chemistry and pathobiochemistry, Ismaninger Str. 22, 81675 Munich, Germany; ^5^„ LMU University Hospital Munich, Department of Psychiatry and Psychotherapy, Nußbaumstraße 7, 80336 Munich, Germany“;^6^„ LMU University Hospital Munich, Medizinische Klinik und Poliklinik II, Marchioninistraße 15, 81377 Munich, Germany“;^7^„ University of Basel, Department of Sport, Exercise and Health, Preventive Sports Medicine and Systems Physiology, Switzerland; ^8^ “Aachen University of Applied Sciences, Heinrich-Mussmann-Str. 1, 52428 Jülich, Germany “;^9^“Technical University of Munich, School of Medicine, Klinikum rechts der Isar, Institute for AI and Informatics in Medicine, Ismaninger Str. 22, 81675 Munich, Germany”;^10^”German Centre for Infection Research (DZIF), Partner Site Munich, Munich, Germany”

Online Data Supplement

**Corresponding author**

Correspondence to Christoph Schmaderer, MD, Department of Nephrology, Klinikum rechts der Isar, Ismaninger Straße 22, 81675 München, Germany. Email [christoph.schmaderer@mri.tum.de](mailto:christoph.schmaderer@mri.tum.de)

|  | **Cohort dependency (PCS/healthy cohort)** | | | |
| --- | --- | --- | --- | --- |
| Predictors | Odds Ratios | CI | Statistic | p |
|  | 0.00 | 0.00 – 0.00 | -3.27 | **0.001** |
| **vFID** | 1.41 | 1.08 – 1.91 | 2.37 | **0.018** |
| **AVR** | 8515756.11 | 975.46 – 480550858470.50 | 3.14 | **0.002** |
|  |  | | | |

**Table E1**

**Table E2**

The general linear regression model shows the relationship between the predictor variables vFID and AVR and cohort dependency. CI indicates “Confidence Interval”. To fit a linear model, residuals were tested for normality.

Models show multivariate linear regression models with SVA parameters AVR, CRAE and CRVE as the depended variables and the interaction terms PCS severity and inflammatory variables CXCL-10, IL-6, ferritin, neutrophils and leukocytes in PCS patients as the two predictor variables. The multivariate model was corrected for age, gender, obesity, arterial (Art.) hypertension and nicotine abuse. CI indicates “Confidence Interval!”.

**Fig. E1: PCS severity score and severity of acute infection and virus variants**

Boxplots show PCS severity score compared between patients with mild acute SARS-CoV-2 infection (white) and patients which were hospitalized (grey) (**a**) and PCS severity score between SARS-CoV-2 virus variants (**b**) Boxplots show values as mean (rectangle) and median (line). Wilcoxon rank sum test was used for nonparametric distributions and Welchs t test for parametric distributions.

Regression model shows potential confounders of RVA and SARS-CoV-2 naïve cohort as a univariate mode for the dependent variables. The multivariate model^b^ was corrected for age, gender, obesity, arterial (Art.) hypertension and nicotine abuse for the dependent variables vmax, CRAE and AVR. The respective R^2^ are shown for the multivariate model^b^ after correction. To fit a linear model, residuals were tested for normality.

**Table E3**

**Table E4**

Parameters of retinal microvasculature in severely affected PCS patients. **a** Boxplot of DVA parameters amax and vmax in 7 moderately affected PCS patients (PCS severity score >10.75 and ≤26.25) (white) and 34 severely affected PCS patients (PCS severity score >26.25,) (grey). Boxplots show values as mean (rectangle) and median (line). To compare groups Wilcoxon rank sum test was used for skewed data and Welch´s t-test for normally distributed data.

P-values are shown for statistical tests comparing moderately (PCS severity score >10.75 and ≤26.25) and severely affected PCS patients (PCS severity score >26.25), t-test was used for normally distributed variables, the χ^2^ test for categorical variables, the Wilcoxon rank sum test for variables with a skewed distribution and the Fisher exact test for binary variables. BMI, body mass index; obesity is defined as BMI > 25 kg/m^2^; hypercholesterolemia is defined as cholesterol > 200 mg/dl.

**Fig. E2**

**Table E4**

**Table E5**

**Table E5**

P-values are shown for statistical tests comparing PCS patients with ME/CFS with PCS patients without ME/CFS; t-test was used for normally distributed variables, the χ^2^ test for categorical variables, the Wilcoxon rank sum test for variables with a skewed distribution and the Fisher exact test for binary variables. ME/CFS, myalgic encephalomyelitis/chronic fatigue syndrome; BMI, body mass index; obesity is defined as BMI > 25 kg/m2. Hypercholesterolemia was defined as cholesterol > 200 mg/dl.

Regression model shows confounders of RVA, PCS with ME/CFS as a univariate^a^ model. The multivariate model^b^ was corrected for age, gender, obesity, arterial (Art.) hypertension and nicotine abuse for the dependend variables AVR and CRAE. The respective R^2^ are shown for the multivariate model^b^ after correction. To fit a linear model, residuals were tested for normality. For one model^a^ F-Statistic was not significant.

**Table E6**

**eTable6**

The multivariate linear regression model shows associations of laboratory parameters with the dependend variables AVR, CRAE, CRVE. D-Dimer was measured in n=36, thrombocytes and ferritin in n=37, TAG, CXCL10, IL8, IL-6, IgG4 and MCP-1 in n=38. The multivariate model^b^ was fitted for age, gender, obesity, arterial hypertension and nicotine abuse. F-statistic was significant for all shown significant models except model^a^.

**Fig. E3**

(**a**) Boxplot shows AVR compared between patients with increased von Willebrand factor (vWF) - activity (>200%, n=2, orange), normal vWF-activity (50-200%, n=34) and reduced vWF-activity (<50%, n=4) Boxplots show values as mean (rectangle) and median (line). ANOVA was used to calculate the p-value and to adjust the p-value the Holms post-hoc test was used. (**b**) Association of AVR and PCS severity score and vWF-activity. Interaction plots show the association between AVR and PCS score for three different levels (+1 standard deviation (SD), Mean and -1 SD) of vWF-activity, selected in the range of the observed data. P_interact_ value indicates interaction between PCS severity score and the inflammatory variable and was computed using a multivariante linear model controlled for confounders age, gender, obesity, arterial hypertension and nicotine abuse.

**Table E7**

Regression model shows confounders of PCS severity and vWF activity as a univariate^a^ model. The multivariate model^b^ was corrected for age, gender, obesity, arterial (Art.) hypertension and nicotine abuse for the dependend variables PCS severity score. The respective R^2^ are shown for the multivariate model^b^ after correction. To fit a linear model, residuals were tested for normality

Association of parameters of retinal microcirculation and PCS severity score with inflammatory parameters. Interaction plots show the association between CRVE and PCS score for three different levels (+1 standard deviation (SD), Mean and -1 SD) of neutrohphils and leukocytes, selected in the range of the observed data. P value indicates interaction between PCS severity score and the inflammatory variable and was computed using a multivariante linear model controlled for confounders age, gender, obesity, arterial hypertension and nicotine abuse.

<x

**Fig. E4**
